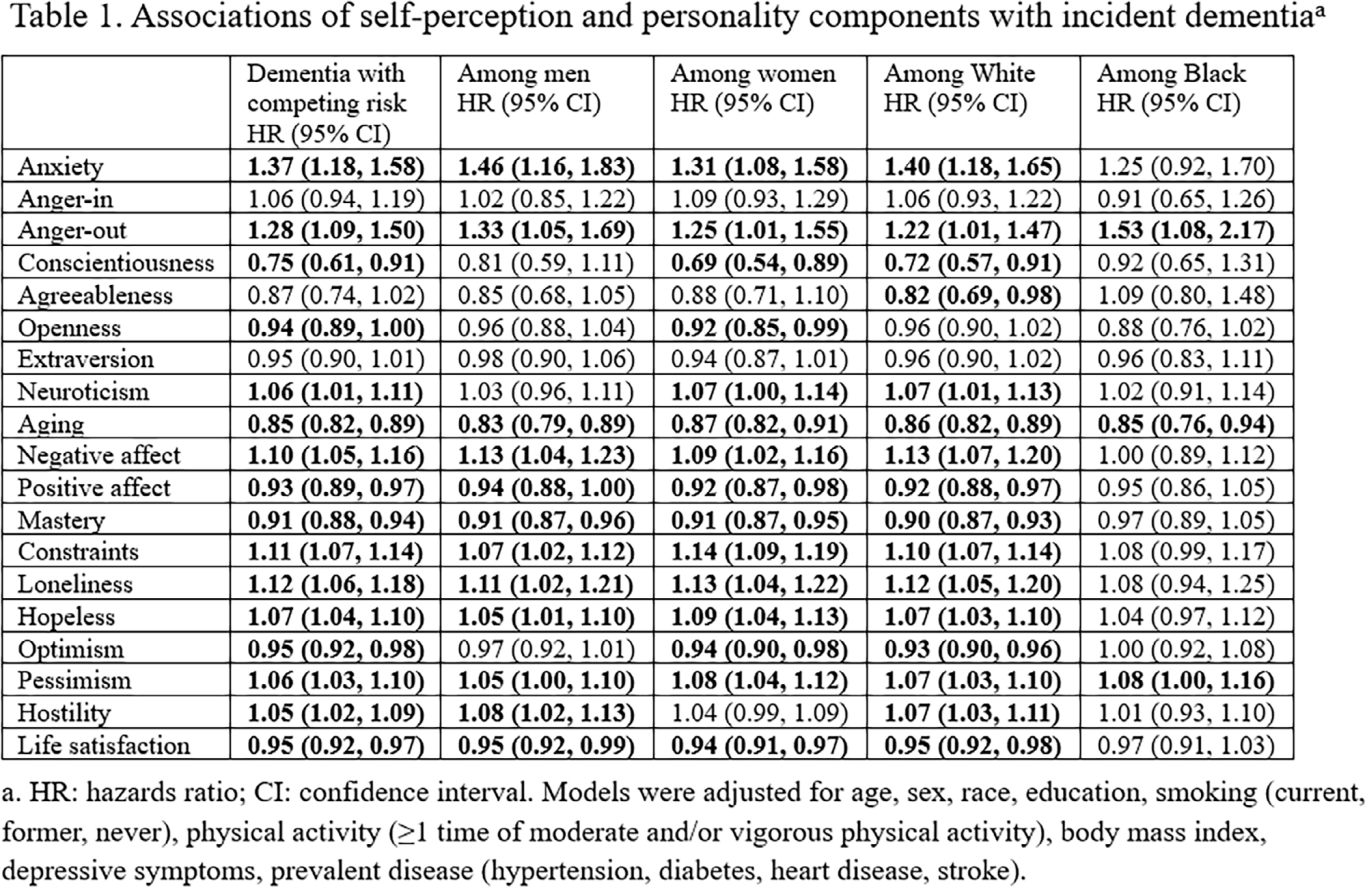# The Associations of Self‐perception and Personality with Incident Dementia: The Health and Retirement Study, 2010‐2020

**DOI:** 10.1002/alz.088518

**Published:** 2025-01-09

**Authors:** Jingkai Wei, Matthew Lohman, Yanan Zhang, Chih‐Hsiang Yang, Anwar T Merchant, Daniela B. Friedman

**Affiliations:** ^1^ University of South Carolina, Columbia, SC USA

## Abstract

**Background:**

Self‐perception and personality may play an important role in the development of dementia through lifestyle modification, social engagement, and mental well‐being. However, evidence regarding the associations of self‐perception and personality with incident dementia has not been well established.

**Method:**

A total of 14,099 participants aged 50 years and older in the Health and Retirement Study (7,335 in 2010 and 6,764 in 2012) were included. They were measured for characteristics of self‐perception and personality using a validated self‐administered questionnaire. This included assessments of anxiety, anger (including anger‐in and anger‐out), five personality traits (neuroticism, extraversion, openness to experience, agreeableness, and conscientiousness), self‐perception of aging, positive and negative affect, sense of control (including perceived constraints and personal mastery), loneliness, hopelessness, optimism and pessimism, cynical hostility, and life satisfaction. Incident dementia through 2020 was self‐reported via questionnaire. Proportional hazards models were used to examine the associations of each component of self‐perception and personality with incident dementia, adjusted for age, sex, race, education, smoking status (current, former, never), physical activity (≥1 time of moderate and/or vigorous physical activity per week), body mass index, depressive symptoms, and prevalent diseases (hypertension, diabetes, heart disease, stroke). Death was considered a competing risk. Subgroup analyses were conducted by sex and race.

**Result:**

After an average follow‐up of 6.9±3.1 years, a total of 590 (4.5%) incident dementia cases occurred. Higher levels of anxiety, anger‐out, neuroticism, negative affect, sense of constraints, loneliness, hopelessness, pessimism, and hostility were associated with a higher risk of dementia. In contrast, higher levels of conscientiousness, openness, positive self‐perception of aging, positive affect, sense of mastery, optimism, and life satisfaction were associated with a lower risk of dementia. Modification of some factors was observed across different subgroups based on race and/or sex (Table 1).

**Conclusion:**

Self‐perception and personality are predictive of incident dementia. Individuals with unfavorable self‐perception and personality profile, indicating a higher risk of dementia, may be monitored for risk reduction.